# Extreme plasma states in laser-governed vacuum breakdown

**DOI:** 10.1038/s41598-018-20745-y

**Published:** 2018-02-05

**Authors:** Evgeny S. Efimenko, Aleksei V. Bashinov, Sergei I. Bastrakov, Arkady A. Gonoskov, Alexander A. Muraviev, Iosif B. Meyerov, Arkady V. Kim, Alexander M. Sergeev

**Affiliations:** 1 Institute of Applied Physics, Russian Academy of Sciences, 603950 Nizhny Novgorod, Russia; 2 Lobachevsky State University of Nizhni Novgorod, Nizhny Novgorod, 603950 Russia; 3 Department of Physics, Chalmers University of Technology, SE-41296 Gothenburg, Sweden

## Abstract

Triggering vacuum breakdown at laser facility is expected to provide rapid electron-positron pair production for studies in laboratory astrophysics and fundamental physics. However, the density of the produced plasma may cease to increase at a relativistic critical density, when the plasma becomes opaque. Here, we identify the opportunity of breaking this limit using optimal beam configuration of petawatt-class lasers. Tightly focused laser fields allow generating plasma in a small focal volume much less than λ^3^ and creating extreme plasma states in terms of density and produced currents. These states can be regarded to be a new object of nonlinear plasma physics. Using 3D QED-PIC simulations we demonstrate a possibility of reaching densities over 10^25^ cm^−3^, which is an order of magnitude higher than expected earlier. Controlling the process via initial target parameters provides an opportunity to reach the discovered plasma states at the upcoming laser facilities.

## Introduction

In the nearest years, several laser-facilities^1–4^ are expected to trigger cascaded production of electron-positron pairs through the processes of quantum electrodynamics (QED)^5–7^. In the early experiments by Burke *et al*.^8^ with pre-accelerated electrons laser fields were used for triggering the generation of hard photons and their decay into pairs. In the presented research, the laser fields are used also for accelerating the produced charged particles so that the field energy feeds the avalanche of pair production. It is possible to draw a simple analogy with optical or microwave breakdown in gases, where primary electrons gain their energy from the fields and subsequently may produce *gas breakdown* giving rise to avalanche multiplication of charged particles. Keeping in mind this analogy, the process of cascade development due to QED processes in vacuum is similar to the breakdown development in gases and thus can be referred to as a *vacuum breakdown*. It should be also mentioned that there is an additional analogy between direct field ionization such as tunnel or multiphoton ionization and pair creation in vacuum when the electric field becomes comparable with the Schwinger field. Although the QED cascade development requires significantly weaker laser fields than the Schwinger field^9–14^, the cascade growth rate^15^ can be sufficiently high to enable extreme scenarios that are remarkable in many respects, ranging from general issues of relativistic plasma dynamics to generation of high fluxes of gamma photons and high-energy particles^16–25^. Thanks to the progress in modelling radiation reaction effects and, more generally, QED effects^14,26–29^, different regimes of particle motion in relativistically strong laser fields with allowance for QED processes have been revealed^7,24,30–39^. The obtained results are a useful tool in investigations of QED cascade development and transparent cascade plasma-field structures in different laser field configurations^7,16,18,25–27,30,33,40–42^. Moreover, development of numerical algorithms^14,43,44^ handling problems arising due to exponential growth of pair number allows simulations of interactions of laser pulses with relativistically dense cascade plasma. Such plasma can be confined in optical traps^22^, and can cause strong absorption of laser radiation^14,16^. However, the self-action of the emerging electron-positron plasma and its ultimate states is still unclear to a large degree.

In the present study we consider reaching an ultimate state of plasma during cascade development in the e-dipole field^45^ which is the utmost case of the colliding beam concept^9,11,13^. Apart from reaching the strongest possible field for a given power of a laser facility, the e-dipole wave provides a remarkably localized field in a volume of much less than λ^3^ and enhances particle localization in a small bulk around the centre by the anomalous radiative trapping effect^46^. We show that with the density growth in this bulk the extreme alternating currents driven by the laser field make the plasma unstable and lead to plasma stratification into thin sheets, where the plasma reaches an extreme state in terms of current and density. At a later stage, they merge and eventually form two aligned sheets. We carry out careful analysis and explain the underlying physics and use 3D QED-PIC simulations^29^ to demonstrate a possibility of reaching this scenario at the upcoming laser facilities with a total peak power of around 10 PW.

## Results

### Vacuum breakdown in an e-dipole wave

**Figure 1:**
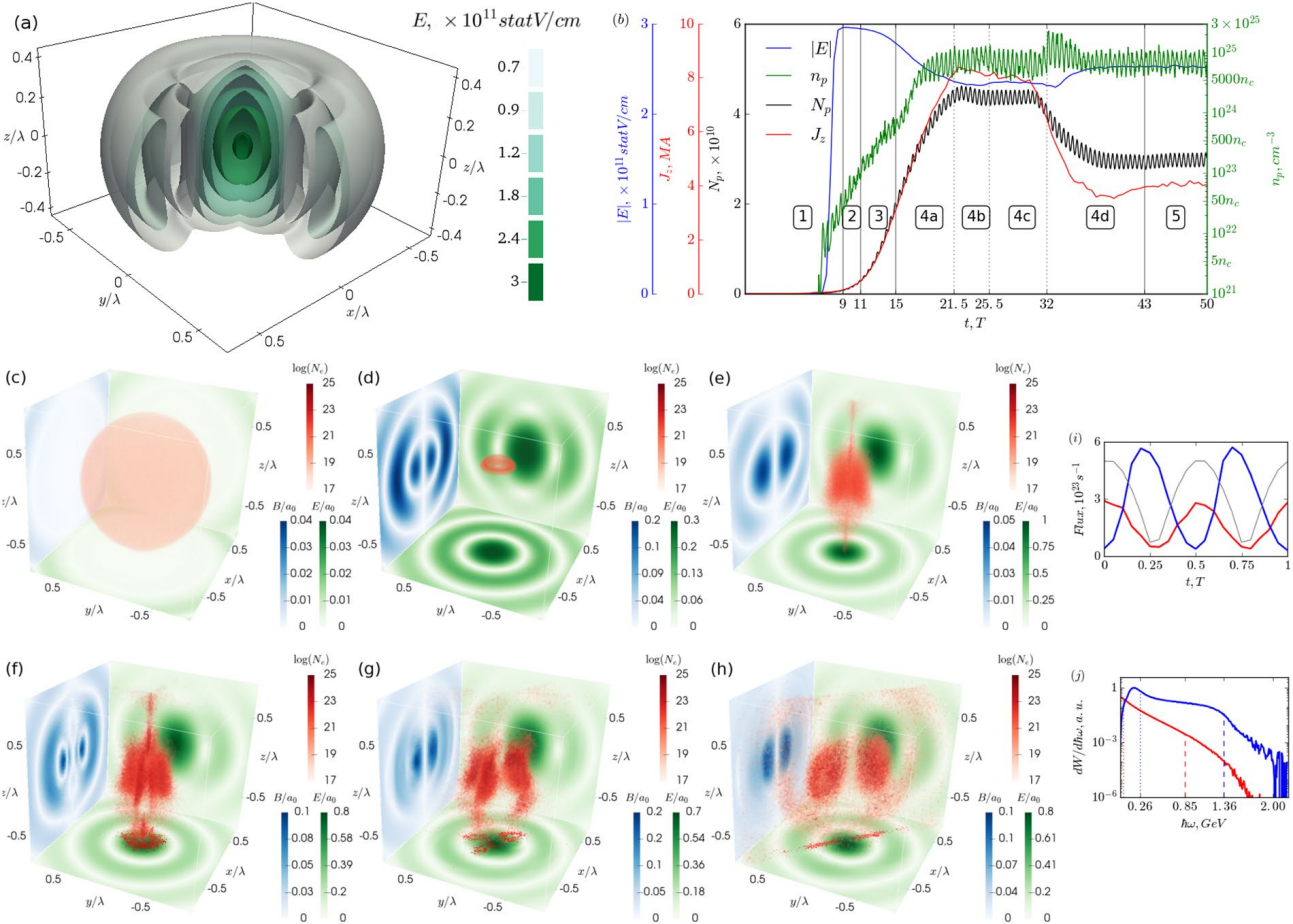
3D PIC simulation of vacuum breakdown in a 10 PW e-dipole wave. (**a**) Contour plot of the electric field of e-dipole wave. (**b**) Timeline of interaction. Main stages are shown: 1 – target compression and formation of standing wave structure; 2 – linear cascade; 3 – nonlinear stage; 4 – development of current instability: 4a – linear stage of current instability and merger to four sheets, 4b,c – merger to three and two sheets, respectively, 4d – relaxation to stationary state; 5 – final stationary state. The curves depict maximum electric field *E* (blue solid line), maximum positron density *n*_*p*_ (green solid line), total current *J*_*z*_ through plane *z* = 0 (red line), number of positrons in a cylinder with diameter and height of *λ* *N*_*p*_ (black line). (**c**–**h**) Plasma-field structure evolution: (**c**) initial plasma distribution; (**d**) target compression; (**e**) linear stage of cascade; (**f**-**g**) current instability development; (**h**) final stationary state. Green and blue surface depict magnetic *B* and electric *E* fields, respectively. Electron density *N*_*e*_ plotted to a logarithmic scale is shown by red. Red contour at the bottom plane depicts plasma contour at plane *z* = 0. (**i**) Temporal structure of emitted positrons (blue), photons (red) with energy exceeding 1 GeV and electric field in the centre (grey) in stationary state. (**j**) Spectra of emitted positrons (blue), photons (red) in stationary state averaged over wave period, dashed lines show maximum energy such that particles with energy exceeding this value possess 1% of total particle energy, dotted lines depict average particle energy.

We start with showing how the extreme states of electron-positron plasma emerge in a particular case of vacuum breakdown governed by an e-dipole wave with a constant total power of 10 PW raised smoothly over one cycle. The result of a 3D QED-PIC simulation of this process is shown in Fig. 1.

Once the e-dipole wave power exceeds *P*_*th*_ = 7.2 PW, rapid pair production enables exponential growth of the number of particles in the small vicinity of the centre (see the Methods section Breakdown threshold). As long as the plasma density is well below the critical value, the back reaction of the plasma onto the field is negligible, and a QED cascade develops in the given fields. This linear stage is well studied for various field structures, such as plane standing waves^10,15,26,42^, several focused laser pulses^9,11^, and an e-dipole wave^17^. An e-dipole wave has a standing-wave-like structure where particle motion is strongly affected by the radiation losses leading to the anomalous radiative trapping (ART) effect^34,46^. In the ART regime the particles oscillate predominantly along the electric field vector gaining energy up to *γ* ~ *a*_0_, here *γ* is the Lorentz factor and *a*_0_ is the dimensionless amplitude of the electric field. The particles form a narrow cylindrical column in the centre with the height of ~0.5*λ* along the *z* axis and the radius of ~0.2*λ* (see Fig. 1(e)), where *λ* = 910 nm is the wavelength of the laser radiation forming the dipole wave.

The nonlinear stage occurs when the pair density exceeds 10^23^ cm^−3^ and becomes comparable with the relativistic critical value *γn*_c_, where *n*_c_ = *πmc*^2^/*(2λe)*^2^ is the critical plasma density for the laser radiation, *c* is light speed, and *e,m* are electron charge and mass, respectively. At this stage, dense electron-positron pair plasma starts to influence the field structure leading to a gradual decrease of the field amplitude, while the field structure remains almost unperturbed. A lower field amplitude implies a lower cascade growth rate, which manifests itself as a gradual slope decrease of the curve describing time evolution of positron density *n*_*p*_*(t)* at *11–*15 *T* in Fig. 1(b).

When the cascade growth rate becomes comparable to the relativistic plasma frequency, an unexpected effect of symmetry breaking of the axisymmetric laser-plasma distribution comes into play due to current instability. The quasistationary plasma-field structure becomes unstable along the azimuthal angle *φ*, which leads to the development of small-scaled density perturbations. Since electrons and positrons moving in opposite directions produce strong axial current, its azimuthal fluctuations generate an alternating radial magnetic field *B*_*ρ*_. The pairs begin to be attracted to *B*_*ρ*_ nodes, thereby locally increasing current density, which, in turn, causes additional growth of *B*_*ρ*_, consequent stronger compression of pairs in the vicinity of the nodes and further growth of current density. Initially, the pair plasma column breaks down into many sheets, which look like narrow rays in the *z* = 0 plane. This process manifests itself as a rapid increase of *n*_*p*_*(t)* slope close to *t* = *15* *T* in Fig. 1(b). Later, due to current interactions, the sheets are attracted to each other forming denser sheets with correspondingly higher currents. After each merger the system relaxes to the new equilibrium state with lower total current and number of particles. Each merger can be distinguished as a local peak in the positron density curve in Fig. 1(b), mergers into four (*t* = *21.5* *T*), three (*t* = *25.5* *T*) and two (*t* = 32 *T*) current sheets are shown in Fig. 1(f,g,h). This effect is most pronounced for the last merger to the final steady state in the form of two aligned sheets. The sheets form a single plane oriented at a random azimuthal angle. This plasma-field structure is stable because it preserves minimum axial symmetry and consists of a minimum number of sheets spaced apart along the azimuth as much as possible, while the electric field hump confines radial particle motion.

### Multi-beam setup

**Figure 2:**
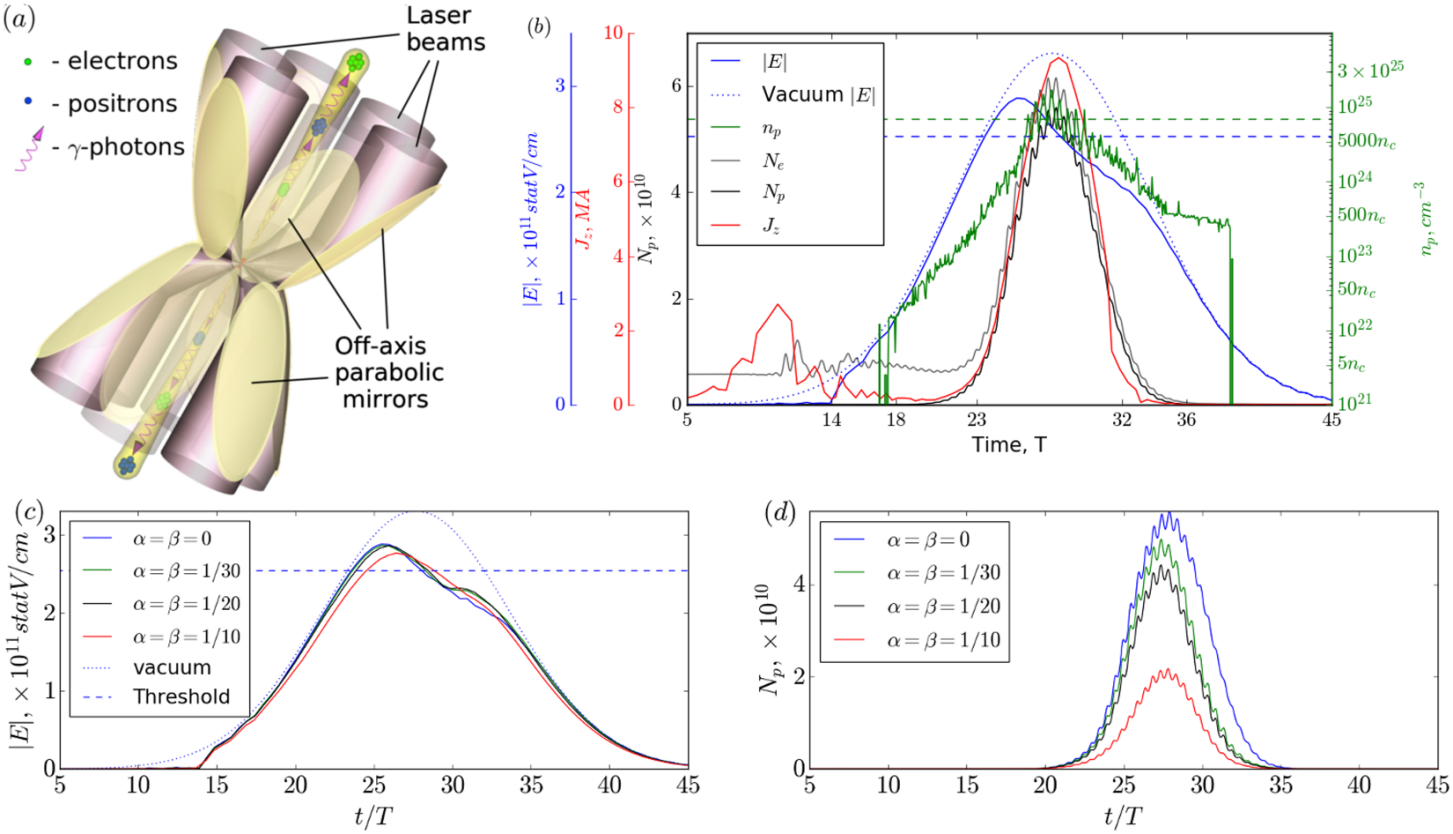
Nonlinear interaction of 30 fs 15 PW two-belt 12–beam lasers with dense plasma target. (**a**) Schematic of the proposed experimental configuration. (**b**) Timeline of interaction. Curves depict the electric field envelope in the centre of the simulation region *E* (blue solid line), envelope of the vacuum electric field *E* in the centre of the simulation region (blue dotted line), field amplitude corresponding to stationary state, specifically, *P*_*th*_(blue dashed line), maximum positron density *n*_*p*_ (green solid line), maximum electron-positron plasma density corresponding to stationary state (green dashed line), the number of electrons in the cylinder with height and diameter equal to *λ* *N*_*e*_ (grey line), the number of positrons in the same cylinder *N*_*p*_ (black line), total current *J*_*z*_ in the *z* = 0 plane (red line). (**c**) Electric field in the centre of simulation region and (**d**) the number of positrons in the cylinder with height and diameter equal to *λ* for different maximum shifts of laser pulse focal point *αλ* and delay of laser pulse *±βT*. The delay and shift for each of 12 laser pulses is randomly set within the specified boundaries.

In experiments the dipole-like wave structure can be mimicked by a number of tightly focused laser pulses. For a fixed power, the field distributions will be close to an ideal dipole wave but with lower maximum field intensity due to a limited aperture compared to *4π* dipole wave focusing. We suggest using a two-belt 12-beam laser system, a schematic of which is shown in Fig. 2(a). First of all, when the number of beams increases, the total power needed to reach desired field intensity decreases, namely, to effectively simulate a 10 PW e-dipole wave we need 35 PW for a 4-beam configuration, 20 PW for a 6-beam configuration and only 12 PW for the proposed configuration using the same f-number optics. Thus, the required power in one beam is about 1 PW, which is already available at the present-day laser facilities. Second, a two-belt configuration allows forming a more optimal field structure in comparison with one-belt configurations, as it covers a larger part of the radiation pattern. Third, this setup still has a reasonable number of beams, taking into account experimental realization issues.

To be more specific about the properties of the extreme state achieved in such a complex geometry, we present the timeline of the nonlinear interaction in Fig. 2(b) based on 3D PIC modelling (see the Methods section Numerical experiment setup). We would like to emphasize that the plasma-field dynamics undergoes the same stages and the structure and properties of the achieved state are similar to those observed in a semi-infinite wave. In contrast to the considered low density case, at the compression stage a dense plasma target is not completely pushed to the centre, and the field is mainly reflected. As soon as the field amplitude becomes high enough to penetrate into the dense plasma (*t~14* *T*), the electric field in the centre grows unevenly and a structure close to the e-dipole wave is formed. Then, the cascade starts to develop leading to a rapid increase in the electron-positron plasma density. In this case, the initial density of the compressed electrons is sufficient to pass quickly to the nonlinear stage of the cascade development, even at the rising edge of the pulse. Initially, a nonuniform intensity distribution together with the high electron-positron plasma density lead to a very fast process of layering the current into a small number of sheets, which ultimately leads to the two-sheet distribution. The pair plasma density exceeds 10^25^ cm^−3^, the value of the electric field is close to the breakdown threshold and a photon flux up to 10^23^ photons per second with energies exceeding 1 GeV is generated during the interaction.

To be close to real experimental conditions, we impose random pulses inaccuracy of two types. The first type is focal spot mismatch, which means that each pulse has its own focal point which lies inside a sphere with radius of *αλ*. The second type of inaccuracy is the time delay of pulse arrival to its focal point, which we set to be *±βT*. Results of numerical experiments with *α = β = 1/10, 1/20, 1/30* show that the observed effect of extreme states formation is stable with respect to small deviations, which is explained by the nature of the current instability. It should be noted that maximum field amplitude, although affected by pulse mismatch in this case, is mainly limited by self-consistent laser-plasma dynamics, which is clearly demonstrated in Fig. 2(c). It can be seen that field amplitude reaches approximately the same values for all pulse mismatches, but at slightly different moments of time. Even for the highest degree of inaccuracy of *α = β = 1/10*, an extreme state is formed although characteristic values such as the number of electrons/protons, corresponding current and density reduce approximately 3 times, as shown in Fig. 2(d).

## Discussion

**Figure 3:**
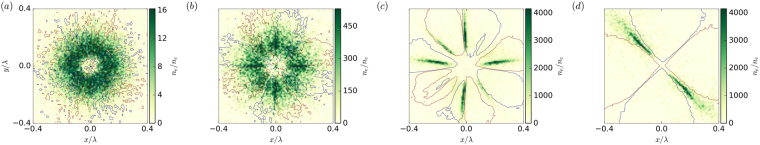
Symmetry breaking in the field of an e-dipole wave. Snapshots of plasma-field structures for a 10 PW e-dipole wave in the z = 0 plane. Contour field panel depicts *B*_*ρ*_ at 0.15 of maximum value, green color shows electron density normalized to the critical value for different moments of time: (**a**) *10 T*, uniform distribution; (**b**) *17.5 T*, multisheet stage; (**c**) *21.5 T*, nonlinear stage of four current sheets; (**d**) *46 T*, final stable distribution of two sheets. For better visualization magnetic field curves are at the level 0.15 of the maximum value, maximum color bar density value corresponds to 0.5 of the actual maximum value.

The most unexpected effect in the formation of electron-positron-pair plasma is that, even in the case of the highest symmetry of incident field configuration, plasma-field structures with essentially lower symmetry are formed as shown in Fig. 3. This process of symmetry breaking can be explained only by the development of instability.

A very intriguing question is: “What are the mechanisms of this instability that violate the symmetry of the laser-plasma interaction?” In general, they can have electrodynamic nature, as a strong plasma-laser field interaction occurs. Paying attention to the fact that the instability is small-scaled with density stratifications along the magnetic field of the laser wave and the entire plasma structures are well localized in the focal volume with dimensions smaller than the laser wavelength, we conclude that this is not the result of nonlinear laser plasma interactions themselves. Moreover, since the pair plasma is actually streams of electrons and positrons moving in opposite directions in the electromagnetic field, current instability can arise even in the given field.

At the linear stage of cascade development, plasma distribution is uniform and *B*_*ρ*_ has a noise-like structure, see Fig. 3(a). When the plasma density approaches the relativistic critical density, the distribution clearly becomes non-uniform, and the radial magnetic field component *B*_*ρ*_ emerges together with density fluctuations, see Fig. 3(b). Since co-directional currents are attracted to each other, at the nonlinear stage of instability the adjacent sheets merge along the azimuth, while radial merging is suppressed by the ponderomotive force. A highly inhomogeneous current structure is formed with several distinct well seen current sheets and the characteristic *B*_*ρ*_ value is of the order of 0.1 of the vacuum field, as shown in Fig. 3(c). Although the field structure is partly modified due to dense current sheets, particle motion is still ART-like and mainly in the plane. Finally, a stable configuration of two sheets at the angle of π from each other is formed; particles during their motion do not cross the *z* axis, as is shown in Fig. 3(d). In this case, the value of *B*_*ρ*_ becomes comparable with the value of the vacuum field. Plasma tends to concentrate at the nodes of *B*_*ρ*_, forming extremely thin structures that computer simulations do not properly resolve. Indeed, doubling the number of cells along the *x* and *y* directions of the simulation box leads to doubled maximum density. It is also interesting to note that the final current direction is not the most apparent one at intermediate stages, which indicates a complex nature of the current dynamics.

**Figure 4:**
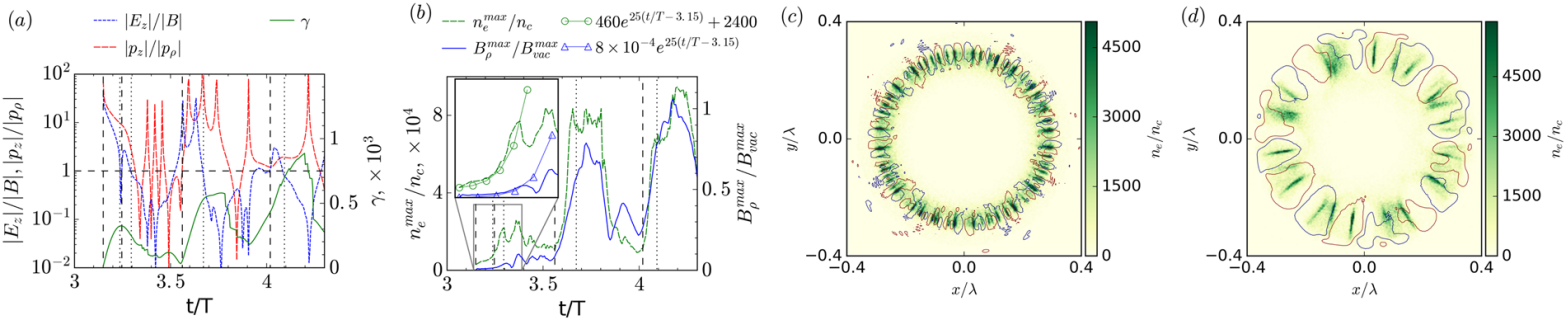
Current instability of electron-positron plasma in the field of cylindrical wave. The electric field amplitude in focus is *a* = 2500. (**a**) Electric field to magnetic field ratio (dashed line), longitudinal *p*_*z*_ to radial *p*_*ρ*_ electron momentum ratio (dash-dotted line), and gamma factor (solid line) along a typical particle trajectory. (**b**) Time evolution of maximum electron density and magnitude of radial magnetic field during development of instability. The horizontal dashed line represents unity level. The time interval when the electric field and *p*_*z*_ exceed the magnetic field and *p*_*ρ*_, respectively, is between the vertical dashed and dotted lines. In the inset the exponential approximation is shown by lines with symbols. The initial perturbation of radial magnetic field and density are equal to ${B}_{\rho }^{0}\approx 8\times {10}^{-4}{B}_{\max }^{vac}$ and ${\tilde{n}}_{0}\approx 460{n}_{c}$. Spatial distribution of the generated radial component of the magnetic field (coloured level curves) and electron density at time instants (c) *t* = *3.48* *T*, (d) *t* = *3.95* *T*. For better visualization the curves are at the $\pm \frac{1}{7}{B}_{\rho }^{\max }$ level.

To get an insight into the physical mechanisms causing instability, we considered a simple two-dimensional case: the interaction of an e-type cylindrical wave with plasma distributed within a cylindrical ring. This setup allowed us to analyse the pure axisymmetric case with uniformity along the *z* axis. In this simplified case, there are only *E*_*z*_ and *B*_*φ*_ laser field components, but the field structure is very close to the field distribution in the central cross-section *z* = 0 of the dipole wave. We performed a PIC simulation of standing cylindrical wave interaction with the plasma ring (see the Methods section Numerical experiment setup). The result of the PIC simulation is shown in Fig. 4.

As simulation shows, during the time interval *3.15* *T* < *t* < *3.3 T* the particles are accelerated along the electric field, increasing mainly the longitudinal component of the momentum and reach the maximum gamma factor *γ*_*max*_ = 300, see Fig. 4(a). Along with this, the azimuthally modulated plasma-field structure emerges from the noise. They grow fast so that at *3.3* *T*, the maximum density becomes an order of magnitude greater than the initial density, and the radial component of the magnetic field becomes 0.1 of the maximum azimuthal component of the standing wave ${B}_{\max }^{vac}$, see the inset in Fig. 4(b). Spatial plasma-field distributions show the same behaviour as in the dipole wave. Initially, many current sheets are generated, but later they merge, see Fig. 4(c,d) and radial and azimuth magnetic fields become comparable. Detailed comparison of Fig. 4(a,b) shows that the perturbations mainly develop when the motion of the particles proceeds along the *z* axis and the axial electric field exceeds the azimuthal magnetic field.

For these time intervals taking into account rapid development of instability, we can derive a simple model of the instability based on hydrodynamics equations coupled with the Maxwell equations. In an unperturbed case the electric field accelerates electrons and positrons in opposite directions, particles move along the *z* axis with velocity ±*v*_0_ (the corresponding gamma factor is *γ*_0_) producing axial current

1 $${j}_{z}=2e{n}_{0}{v}_{0}.$$

There is no azimuthal motion and for estimations it is assumed that the radial velocity of the particles is much less than ${v}_{0}$ and can be neglected. While the *j*_*z*_ structure is axially symmetric, current generates only azimuthal magnetic field. However, such a symmetric structure is unstable and instability leads to generation of other field components and excites other motions. We assume that the modulation of plasma density, current, radial magnetic field and azimuth velocity may be written in the form

2 $$\tilde{n},\,{\tilde{j}}_{z},\,{B}_{\rho },\,{v}_{\phi } \sim {e}^{{\rm{\Gamma }}t+il\phi }$$

respectively, where *Γ* is instability growth rate, *l* is integer and *φ* is azimuthal angle. Assuming that the instability growth rate is greater than the field frequency, we suppose that the distributions of the plasma and the electromagnetic field are approximately constant in time. Maxwell equations show that perturbation of axial electric field and azimuth magnetic field can be neglected in comparison with ${B}_{\rho }$, if $l\gg {\rm{\Gamma }}T/(2\pi )\gg 1$ for relativistically dense plasma. This inequality is confirmed by the computer simulation demonstrating a lot of current sheets at the initial stage of the instability (see Fig. 4(c)). From Maxwell equations it also follows that the plasma density perturbation $\tilde{n}$ leads to generation of a radial component of magnetic field

3 $${B}_{\rho }=\frac{i8\pi e\rho {v}_{0}\tilde{n}}{lc}$$

Taking into account the condition imposed on *l*, the motion equations allow us to state that the modulation of axial current is ${\tilde{j}}_{z}\approx 2e\tilde{n}{v}_{0}$ and perturbations of the axial and radial velocities are negligible in comparison with ${v}_{\varphi }$. Since the azimuthal magnetic field can be neglected, we omit the radiation reaction force. Validity of this assumption is confirmed by the absence of significant energy leaps due to the photon emission in the considered time interval; see the green solid line in the time interval *3.15 T* < *t* < *3.3 T* in Fig. 4(a). From the motion equation

4 $${\rm{\Gamma }}m{v}_{\phi }{\gamma }_{0}=\frac{e}{c}{v}_{0}{B}_{\rho },$$

we see that *B*_*ρ*_ is responsible for azimuthal motion with velocity ${v}_{\phi }$. The pairs are attracted to the nodes of *B*_*ρ*_, and density perturbations can be obtained from the continuity equation

5 $${\rm{\Gamma }}\tilde{n}+i\frac{l}{\rho }{n}_{0}{v}_{\phi }=0.$$

Thus, not only $\tilde{n}$ and correspondingly ${\tilde{j}}_{z}$ excite *B*_*ρ*_, but *B*_*ρ*_ increases plasma density perturbation as

6 $$\tilde{n}=\frac{-il{B}_{\rho }e{n}_{0}{v}_{0}}{{{\rm{\Gamma }}}^{2}\rho m{\gamma }_{0}c}.$$

As a result, the self-excited process of the current instability occurs with the growth rate

7 $${\rm{\Gamma }}=\frac{{\omega }_{p}}{\sqrt{{\gamma }_{0}}}\frac{{v}_{0}}{c}\cong \frac{{\omega }_{p}}{\sqrt{{\gamma }_{0}}},$$

where ${\omega }_{p}=\sqrt{\frac{8\pi {e}^{2}{n}_{0}}{m}}$ is the plasma frequency taking into account contribution of electrons and positrons.

For the realistic parameters *n*_0_ = *2400n*_c_ and *γ*_0_ ≈ *γ*_*max*_*/2* = 150, the estimate of the instability growth rate *Γ* ≈ *25/T* corresponds quite closely to the results of numerical simulations shown in Fig. 4(b). The important consequences confirming the derived instability model are the following. First, the increment does not depend on the wave number of the disturbance, which indicates a possibility of growth of perturbations of different scales, which is actually clear from the Fig. 4(c). Second, due to fixed radial particle motion in the laser field, plasma decomposes into sheets along the azimuth angle rather than into filaments. Third, the increment is anomalously large and formally close to the growth rate of the Weibel instability^47^, but unlike the latter, the current sheets are purely quasi-neutral, which is in full conformity with the simulations.

**Figure 5:**
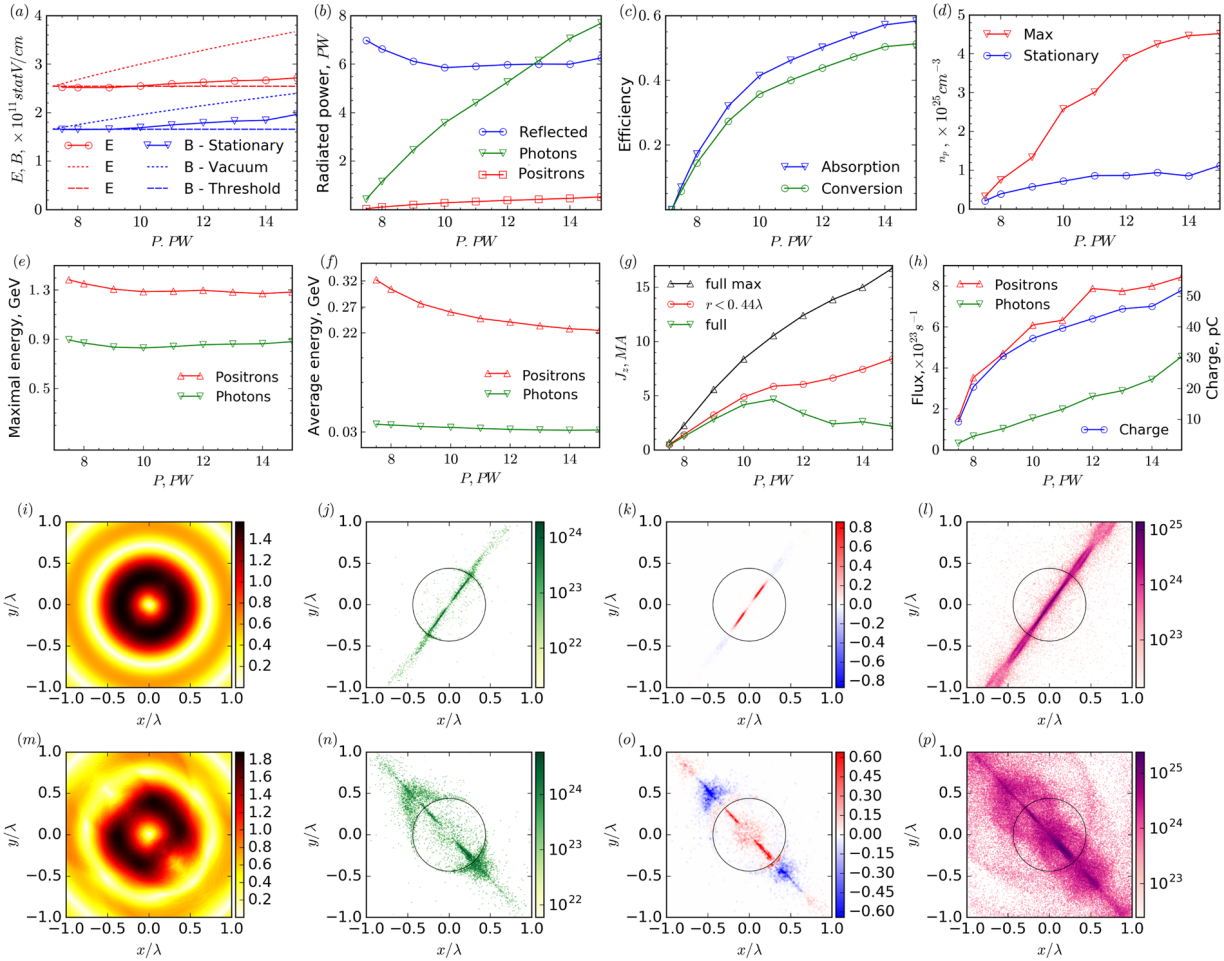
Properties of stationary state *vs* e-dipole wave power. (**a**) Maximum electric (solid red line) and magnetic (solid blue line) field in stationary state. Dotted lines represent vacuum fields scaling as *P*^*1/2*^, dashed lines show electric and magnetic field values corresponding to *P*_*th*_. (**b**) Energy balance in stationary state: reflected energy (blue line), energy transferred to photons (green line) and positrons (red line). (**c**) Energy absorption efficiency (blue line) and conversion to gamma photons (green line). (**d**) Pair plasma density in stationary state (blue line) and maximal value at transient nonlinear stage (red line). (**e**) Maximum energy of positrons (red line) and photons (green line). (**f**) Average energy of positrons (red line) and photons (green line). (**g**) Current *J*_*z*_ in *z* = 0 plane in stationary state: total current (green line), current in the central antinode region (radius *r* < 0.44*λ*) (red line) and maximum current at all stages (black line). (**h**) Total charge of a single positron burst (blue line), averaged over period electron (red line) and photon (green line) flux with energy exceeding 1 GeV. (**i**–**p**) Structure of positron density and currents in stationary state at *z = *0 plane for (**i**–**l**) 8 PW and (**m–p**) 15 PW. From left to right: (**i**,**m**) magnetic field (×10^11^G), (**j**,**n**) positron density (cm^−3^) plotted to a logarithmic scale, (**k**,**o**) current density (×10^16^ A/cm^2^), (**l**,**p**) photon density (cm^−3^) plotted to a logarithmic scale.

Self-consistent plasma-field states are the result of nonlinear interaction of the incident wave with the self-generated pair plasma through QED cascades which are dependent on laser wave power. These states are stationary on the average over the laser cycle, i.e. the average cascade growth rate is zero and the total number of particles does not change. Nevertheless, within the laser period the density changes and particle escape from the focal region is compensated by production in the intense field. The structure of steady state fields is close to a dipole wave, so, according to the notion of a vacuum breakdown threshold (see The Methods section Breakdown threshold), the steady state field amplitude has to be close to the threshold value. Simulations show that for power below 10 PW, both electric and magnetic fields get stabilized slightly below the threshold value, as shown in Fig. 5(a). At higher power, the plasma structure changes, as shown in Fig. 5(i–p), which leads to a slight deviation from the threshold. For lower powers, the plasma distribution is well localized inside the central part within the maximum of the electric field retaining sub-wavelength plasma size, but for higher powers a fraction of charged particles is pushed to the electric field minimum and this distribution becomes comparable to the wavelength. These particles have low energy, but their amount increases with power, which leads to a drop in average electron energy, see Fig. 5(f). At the same time, the maximum energy, as shown in Fig. 1(j), follows the dependence of electric field on power and remains nearly constant and close to 1 GeV level for both positrons and photons, see Fig. 5(e). Averaged over the laser period, plasma density in these structures reaches values up to 10^25^ cm^−3^ (see Fig. 5(d)), exceeding relativistic critical density. It should also be noted that current sheet distribution may be very narrow, as all particles are attracted to a single plane, and the maximum density values may be limited by grid resolution. The number of positrons *N*_*p*_ in these sheets grows from 10^10^ for 8PW to 10^11^ for 15 PW. These particles oscillating along the *z*-axis create *J*_*z*_ current, the total current in the *z* = 0 plane for different powers is plotted in Fig. 5(g). The maximum values of current and pair density are observed at the current instability stage and can be as high as 17 MA and 4.5 × 10^25^ cm^−3^ for 15 PW, respectively, see Fig. 5(d,g). During the development of instability the number of current sheets decreases, as well as the total number of particles, and the system relaxes to lower currents. At low power in a stationary state, particles are mainly located within the central antinode region of electric field (radius *r* < 0.44 *λ*). The current in this region increases with power increase as the number of particles rises. For higher powers, the particles are pushed to the next antinode region (0.44 *λ* < *r* < 0.97*λ*) where the electric field as well as the generated current have opposite directions as compared to the central region, see Fig. 5(n), thereby decreasing the total current *J*_*z*_ from 5 MA for 10 PW to approximately 3 MA for 15 PW, see Fig. 5(g).

The generated electrons and positrons have a high enough probability to escape the focal region, for rough estimates we assume it to be 0.1. The electrons and positrons are emitted in the form of bursts with duration an order of *τ*_*b*_ ≈ *¼T* in opposite directions along the electric field (*z*-axis), so that in each direction the burst period equals *T*. Photons are emitted symmetrically in both directions along the *z* axis with a period of *T/2* with a *π/2* phase shift relative to the particle bursts, see Fig. 1(i). The peak fluxes of both charged particles and photons with energies exceeding 1 GeV grow from a value of about 10^23^ for a laser power of 8 PW to 10^24^ for 15 PW as shown in Fig. 5(h). Based on the number of pairs in steady states, the total flux of pairs and photons can be estimated to be 0.1 *N*_*p*_/*τ*_*b*_ ≈ 10^25^–10^26^ s^−1^ for power from 8 to 15 PW, which is in good correspondence with simulations. Correspondingly, the total charge in one electron (positron) burst is about 0.1*eN*_*p*_ ≈ 1–10 nC; however, particles with energies exceeding 1 GeV carry about tens of pC. Thus, by separating electrons and positrons in space, for example, by means of an external magnetic field, it is possible to control the total charge by choosing the duration of the laser pulse. For example, for the 30 fs duration of incident pulses, a total charge of GeV particles can be up to 0.5 nC. The energy acquired by particles from incident waves is much less than the laser energy transformed into gamma radiation, see Fig. 5(c). The conversion efficiency of laser energy into gamma photons approaches 55% for higher power and the absorption in high-density pair plasma may exceed 60% as shown in Fig. 5(d).

**Figure 6:**
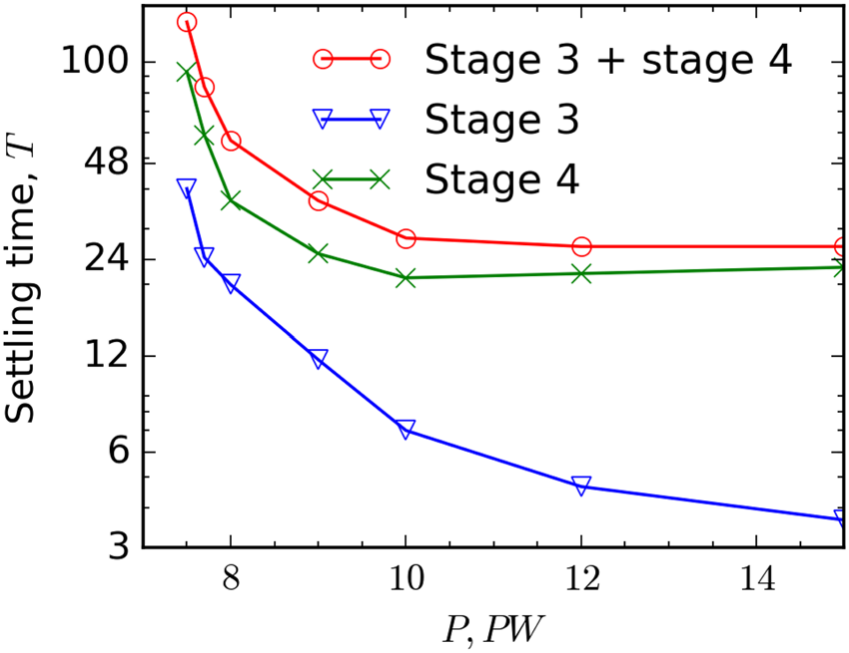
Time scale of transition to stationary state. Time for transition from the end of the linear stage to current instability stage – stage 3 in Fig. 1(b) (blue line), the duration of the instability stage – stage 4 in Fig. 1(b) (green line) and time for transition from the end of the linear stage to steady state – stages 3 and 4 in Fig. 1(b) (red line).

Another important issue is achieving such extreme states of matter and antimatter in experiments. The characteristic time of the transition to a stable steady state is shown in Fig. 6. For a power slightly above *P*_*th*_, this time can exceed 100 optical cycles, which is quite natural, as the cascade growth rate is low. For higher power it stabilizes at a level of about 25 laser cycles, which still exceeds the feasible duration of the laser pulse with a required power of 10–15 PW. This saturation can be explained based on the timeline shown in Fig. 1(b). Laser-plasma dynamics undergoes several stages, but the major part of this time is spent on the stage of the current sheets merger and relaxation to the final steady state of two aligned sheets (stage 4). This transition occurs under the conditions that weakly depend on incident power, because at the instability stage the electric field drops down to a near-threshold level.

At first sight, such a long transition time looks non-optimistic on the way to realizing extreme states of matter and antimatter in experiments. We can argue this by two important considerations. First, the transient quasistationary stage of the current multi-sheet formation with the most extreme states can be achieved within 3–5 periods, even at a moderately high power of 10 PW. Second, development of current instability involves breaking of the axial symmetry, when the initially uniform distribution becomes a very narrow flat distribution. This process develops even for an ideal dipole wave without any explicit inhomogeneity, but the initially non-uniform angular distribution of intensity or plasma can significantly accelerate the process of breaking symmetry and, thus, reaching a final steady state. This simple reason makes the multi-beam setup even more preferable, since an *n*-beam intensity distribution produces *n* seed sheets.

Another way to shorten the duration of the transition to a stationary structure is to control plasma seed parameters. For attaining extreme pair plasma states on a timescale of several optical cycles, we need to pass to the nonlinear stage of laser-plasma interaction as quickly as possible. For this it is necessary to consider targets with solid-state densities. First, such targets allow shortening the linear stage of the cascade. Second, heavier and slower ions reduce fast electron escape, decreasing *P*_*th*_, therefore the cascade can develop during a larger part of the laser pulse. Third, dense targets can function as a plasma mirror^48^. These targets have to reflect incident waves while instantaneous power is less than *P*_*th*_. In this case, the field penetrates into the plasma over skin depth and does not cause abundant particle escape, thus plasma density should be of about relativistic critical value *n*_*c*_*γ*, where *γ* corresponds to *P*_*th*_. Since incident waves compress targets and ions lead to the decrease of the vacuum breakdown threshold, it suffices to consider initial densities of about 10^22^ cm^−3^ according to PIC simulations.

Summarizing, we have demonstrated that extremely dense electron-positron pair plasma can be generated using currently available petawatt lasers. This can be reached in a multi-beam configuration of the laser system setup, particularly in the optimal case by combining 12 laser beams, for which the field structure is very close to the ideal case of converging e-dipole wave allowing the smallest focal volume to be obtained. In this λ^3^-regime pair plasma represents interpenetrating streams of extreme densities of electrons and positrons and, correspondingly, large currents, energies of particles and gamma photons lie in the GeV range. Such an object of matter and antimatter is of great interest for fundamental particle physics as well as for astrophysics. We believed that obtaining pair densities of about 10^24^–10^25^ cm^−3^ in laboratory could provide a unique state of dense matter and antimatter.

## Methods

### Breakdown threshold

Tight focusing of laser pulses leads to an increase of electric field strength and enhancement of electron-positron pair generation, however particles get an opportunity to escape the interaction focal region, thus reducing the cascade growth rate or even leading to expansion of the seed target. For an e-dipole wave, the main parameter is its power. If the power exceeds the petawatt level, a special ART regime emerges and traps particles. In this case they can leave the focal region mainly along the electric field, losing the opportunity to create pairs. The growth of power reduces particle escape due to trapping and intensifies pair creation. For a continuous wave, this ensures a power threshold at which particle losses are compensated by pair creation. To determine this threshold, we model the linear stage of the cascade in the dipole wave using the PICADOR PIC-code. As a seed, we consider a spherical hydrogen-like target with a radius of 1.5 wavelengths centred in focus and a density of 10^16^ cm^−3^ corresponding to 1000 real particles. At each timestep we count the number of pairs in the focal cylinder with a radius of 0.5 wavelength and height of 1 wavelength, which contains nearly all trapped particles. Their number averaged over a half-period of the wave grows or decreases exponentially. For near threshold powers of 6.5 PW and 8 PW, the total cascade growth rates including pair creation and escape are −0.2/*T* and 0.21/*T*, respectively. Linear interpolation shows that the approximate threshold is 7.2 PW.

### Numerical experiment setup

Two different numerical experiment setups were used for modelling e-dipole wave. The first one is a semi-infinite wave with a rapid front to study characteristics of extreme plasma states for a fixed wave power; the second setup employs Gaussian pulse shape with 30 fs duration to model a 12-beam experimental setup. In both setups laser radiation with a total power of 7.5–15 PW interacted with a hydrogen-like target with 1.5 μm radius. In the first case, the target density was changed in the range from 10^15^ to 10^19^ cm^−3^depending on power to ensure the presence of the linear stage of cascade. In the second case, the density was chosen so that the nonlinear stage was reached at the rear front of the pulse and corresponded to near solid densities of 10^22^ cm^−3^. 12-beams with an f-number of 1.2 with a total power of 15 PW, which roughly corresponds to an ideal e-dipole wave of 13 PW, interacted with a plasma target with a radius of 1 wavelength and a density of 10^22^ cm^−3^. The influence of ionization as well as ion motion on laser-plasma dynamics can be important, especially in case of high-Z elements^49,50^, as the process of triggering the electron-positron cascade can be governed by a proper choice of target parameters. However, for the case of interest we confined ourselves to solid hydrogen targets, assuming that it will be fully pre-ionized already at the leading edges of the pulses.

Numerical modelling was performed using Particle-in-Cell codes PICADOR^51^ and ELMIS^29^. The main difference between these two codes is that FFT-based code ELMIS does not have spatial numerical dispersion as compared to the conventional FDTD method used in PICADOR. FFT based solver was used to ensure that small spatial inhomogeneity imposed by numerical dispersion in such an axisymmetric setup is not a key reason for rapid current instability development. The results of simulations with both codes were qualitatively and even quantitavely similar to a high degree, taking into account random nature of the layering process.

A cubic grid with size of 4 μm × 4 μm × 4 μm and number of points of 512 × 512 × 512 was used in simulations. Due to the Courant stability criterion for the FDTD the time step in PICADOR code was chosen to be 0.01 fs. ELMIS code tolerates higher time steps, but we used the same values for consistency. We used the wavelength of 0.9 μm in conformity with the characteristics expected in the XCELS facility^3^, but qualitatively the dynamics will be close for the setups with 0.8 μm wavelength. For the chosen wavelength spatial and temporal resolutions were approximately 115 and 300 steps per wavelength and period, respectively. For particle push the well-known Boris pusher^52^ was used together with Esirkepov scheme^53^ for current deposition. Special attention was paid to correct modelling of electron-positron cascade development. For this a special Adaptive Event Generator module^29^ was used with separate particle resampling for different particle types. This module has quite sophisticated algorithms, because it has to deal not only with correct modelling of pair and photon production, but these processes must be modelled correctly under conditions when density grows by several orders of magnitude within one period. This is achieved by using novel particles resampling techniques, when at a given step the particle ensemble is replaced by a smaller one with higher particle factors, and special procedures, including time step subdivision, allowing correct modelling of micro-avalanches within a single time step.

We also performed a 2D PIC simulation with the following parameters. The square box with size of 2 μm × 2 μm and number of points 512 × 512 was used with spatial and temporal resolution 230 and 768 steps per wavelength and period, respectively. Standing cylindrical wave with amplitude *a* = 2500 in relativistic units interacted with electron-positron plasma having density *n*_*0*_ = 2400 *n*_*c*_ located within 0.17*λ* < *ρ* < 0.27*λ*, which correlated with the range of radial oscillation in ART regime. The chosen density value corresponded to the beginning of the current instability stage in the 10 PW dipole wave. The initial moment of the interaction was when the magnetic field was zero at the time *t* = 3.15 *T*. For simplicity, pair generation was switched off.
